# Real-time text message surveys reveal student perceptions of personnel resources throughout a course-based research experience

**DOI:** 10.1371/journal.pone.0264188

**Published:** 2022-02-18

**Authors:** Alyssa N. Olson, Sehoya Cotner, Catherine Kirkpatrick, Seth Thompson, Sadie Hebert

**Affiliations:** Department of Biology Teaching and Learning, University of Minnesota, Minneapolis, Minnesota, United States of America; City University of New York, UNITED STATES

## Abstract

Course-based research experiences (CREs) are designed to engage students in authentic scientific experiences that are embedded into a standard curriculum. CREs provide valuable research experiences to large numbers of undergraduate biology students, however, CRE implementation can require many personnel. Because limited personnel may be a barrier to widespread CRE implementation, our goal was to discover which personnel students valued throughout a CRE and the ways they were valuable. We investigated students’ perceptions of personnel resources throughout a semester-long CRE using two survey approaches. Using a text message survey administered multiple times per week, real-time data was collected about which personnel resource students perceived to be the most helpful. Using a web-based survey administered five times throughout the semester, retrospective data was collected about how often students used each personnel resource and how helpful students perceived each personnel resource to be. Graduate teaching assistants (TAs) were consistently selected as the most helpful personnel resource by the majority of respondents throughout the semester, with most respondents describing graduate TAs providing project-specific feedback. Although less frequently, undergraduate TAs were also consistently selected as the most helpful personnel resource. Respondents described undergraduate TAs providing project-specific feedback, general feedback, and project-specific resources. Data from the retrospective, web-based survey largely mirrored the real-time, text message survey data. Throughout the semester, most respondents reported using graduate TAs “Often” or “Always” and that graduate TAs were “Very” or “Extremely” helpful. Throughout most of the semester, most respondents also reported using undergraduate TAs “Often” or “Always” and that undergraduate TAs were “Very” or “Extremely” helpful. The results of this descriptive study underscore the importance of graduate and undergraduate TAs in the development and implementation of CREs, emphasizing the need for departments and course coordinators to be intentional in planning TA training that prepares TAs to fulfill their critical role in CRE implementation.

## Introduction

Numerous biology education stakeholders have called for evidence-based teaching and learning in undergraduate biology classrooms. One element of these calls includes engaging all undergraduate students in research experiences in their courses [1, 2]. For example, a 2012 report by the President’s Council of Advisors on Science and Technology stressed the importance of improving biology education at the college level to increase the number of STEM graduates [3]. A core recommendation in this report was to “advocate and provide support for replacing standard laboratory courses with discovery-based research courses”. Collectively, these and other recommendations [4] have led to the implementation of course-based research experiences (CREs) in undergraduate biology courses. While CREs can consist of slightly different elements [5, 6], most include a discovery-based, hands-on research experience embedded within a course, ranging from a few weeks [7, 8] to a full semester in length [9].

CREs provide solutions to many of the limitations of traditional research apprenticeships while retaining the benefits of participating in research. Research experiences for undergraduate students have traditionally been in the form of summer programs or research apprenticeships, which often have barriers that prevent many students from being able to partake in these programs. For example, not all students may be able to participate due to time limitations or financial constraints, such as needing to spend time outside of class caring for family and/or working a paid job rather than an often-unpaid research apprenticeship. Additionally, these opportunities are limited in number, programs are often highly selective, and many students may be unaware these opportunities exist [2, 3, 10]. In contrast, CREs provide research opportunities for a larger number of undergraduate students, especially lower-division students [11, 12], and students earn course credit for their participation. Moreover, student participation in CREs also results in many of the same positive outcomes associated with participating in a research apprenticeship, including gains in content knowledge, scientific thinking, science process skills, interest in science and graduate education, and persistence in STEM [11, 13–15]. In addition to improving academic outcomes, CREs also provide non-cognitive benefits to students including self-efficacy development, positive views towards laboratory group work, increased development of science identity [16], greater increases in both career and intrinsic motivation compared to their non-CRE counterparts [15], and increased confidence in their ability to carry out laboratory tasks [17].

While CREs provide many benefits to students, continued CRE operation can be challenging due to ongoing space, financial, and personnel requirements. Access to laboratory space and the costs of research supplies or equipment are often cited as barriers to CREs [18–21] and various solutions to these challenges have been proposed including establishing collaborative partnerships to share space, supplies, or equipment [19, 20] or obtaining additional funding [20]. Beyond space and cost limitations, another barrier to continued CRE operation is the personnel-intensive nature of CREs, which often require more time and effort from faculty and staff and/or more personnel than non-CRE courses [20, 21].

Various course personnel can be involved in the operation of the CRE; these include principal investigators (PIs), research or academic mentors, instructors, teaching assistants (TAs), course directors or coordinators, or laboratory staff. These personnel may have unique or overlapping roles, and their roles may depend on the research project, class size, institutional or course support structures, or other factors. Because CREs engage students in different aspects of the process of science throughout a semester (e.g., development of a research question and/or methodology, data collection and/or analysis, presentation of results), students may need different types of support at different stages of their research project. For example, some course personnel may primarily provide input in the early stages of the research process, such as when students are developing research questions and hypotheses or are designing experiments. Other personnel, who are in the laboratory more frequently, may provide consistent support throughout the research experience as students encounter problems while conducting their experiments. Given that limited personnel resources may be a barrier to widespread CRE implementation, the goal of this study was to understand:

1. Which personnel resources were the most valuable to students during the CRE?
2. In what ways were the personnel resources valuable to students?
3. Were different personnel resources more valuable to students at different times during the CRE (e.g., during the project planning at the beginning of the semester vs. during the presentation of results at the end of the semester)?

To answer these questions, we surveyed 57 biology majors participating in a semester-long CRE using a brief text message survey two or three times per week and a longer web-based survey five times throughout the semester. This novel application of the text message survey method allowed us to collect fine-grained data about students’ perceptions of personnel resources in real-time, while the web-based survey allowed us to collect additional information retrospectively, allowing for a more holistic understanding of students’ personnel resource use throughout a CRE. Our primary aim was to characterize the nature of CREs, with respect to the human resources used by students, in hopes of identifying the degree to which personnel demands are a tractable barrier to CRE implementation.

## Materials and methods

### Course

**Fig 1:**
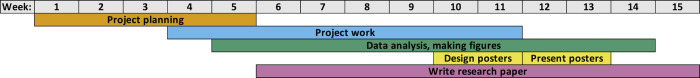
Research project timeline. The research project timeline shows the research project activities and the corresponding weeks of the semester in which they occur.

*Foundations of Biology for Biological Sciences Majors*, *Part II Laboratory* is the second course in an introductory biology laboratory course sequence that is required for all College of Biological Sciences (CBS) majors at the University of Minnesota [9]. In this course, students design and perform research projects in one of five research areas—computational microbiology, environmental toxicology, global change ecology, microbial evolution, or zebrafish microbiome (see Box 1 for research area descriptions). Students working in the computational microbiology or global change ecology research areas perform computational research projects, while students working in the other three research areas perform wet-lab research projects. Within each laboratory section all research projects are focused within a single research area. Sections meet once per week for two hours; these meetings are led by a graduate TA, with an undergraduate TA to assist. Working in small groups of three to six, students develop plans for their research projects and refresh relevant skills during weeks 1–5, carry out their project work in weeks 4–11, design group research posters in weeks 10–11, and present their posters in weeks 12–13. In addition, students individually write research papers (beginning in week 6) that are due in week 15 of the semester (Fig 1). The majority of students’ project work and writing occurs outside the class sections; in class, they give updates about their project, discuss relevant readings, receive instruction related to data analysis and writing, and check in with their group members.

**Box 1. Research area descriptions**

**Computational microbiology:** Students in this area use human gut microbiome data from previously published studies to address new hypotheses; often they compare microbial diversity between different subpopulations of subjects and/or relate microbial diversity metrics to other available data about the subjects [9].

**Environmental toxicology:** Students in this area evaluate the effects of chemical treatments or environmental manipulations on aspects of zebrafish development, physiology, and/or behavior to address hypotheses about the effects of environmental contaminants.

**Global change ecology:** Students in this area use publicly available datasets to address their hypotheses about the effects of anthropogenic changes on ecological systems such as oceans, forests, grasslands, and freshwater lakes [22].

**Microbial evolution:** Students in this area use *Pseudomonas fluorescens* strains that have undergone adaptive radiation or directed selection to evaluate hypotheses about relative fitness under different experimental conditions, evolutionary trade-offs, population diversity, or other properties of the bacterial strains.

**Zebrafish microbiome:** Students in this area evaluate the effects of chemical or other treatments on the composition and diversity of the zebrafish gut microbiome. They also evaluate the effects of their treatment on the properties of bacterial strains isolated from the fish, with the goal of relating population-level changes to the effects on individual members of the population.

### Personnel resources

#### Principal investigators (PIs)

PIs provide context and help decide the overall scope of students’ work when designing the research area, through discussions with the course directors, the research mentor, and lab staff. Sometimes PIs directly answer questions from students, but most often they serve as a resource for the research mentor.

#### Research mentors

Research mentors are postdoctoral scholars or senior graduate students who support students and graduate and undergraduate TAs in their research area. They participate in TA training in the week before the semester begins and attend weekly meetings with the graduate TAs throughout the semester. Research mentors also attend each section (in their area) two to three times during the semester and/or meet with groups of students outside of class; through these interactions, mentors help students refine their research questions and project plans, as well as troubleshoot issues that arise during project work, particularly when graduate or undergraduate TAs lack the relevant specialized knowledge. Research mentors also provide informal feedback to students and support them as an expert in their area who is interested in the work they are doing.

#### Graduate TAs

Graduate TAs lead the weekly section meetings, provide instruction and guidance to students, and grade most of the course assignments. They participate in weekly TA meetings with the course directors, research mentor, and lab staff (when relevant). These meetings include discussions about the group projects that semester, the instructional content, and pedagogical strategies; they provide ongoing training for the TAs and a channel for regular communication. Graduate TAs are assigned to specific lab sections for the duration of the semester.

#### Undergraduate TAs in section

Undergraduate TAs in section assist the graduate TA primarily by checking in with each student group and answering questions or offering advice; they also provide instruction occasionally and grade a few assignments. In sections where students are doing wet-lab work, the section TAs are drawn from the group of undergraduate TAs who staff the lab. The section TAs have direct “hands on” experience in the research area from having taken the course; they help students with practical skills and with navigating the research process. Undergraduate TAs in section are assigned to specific lab sections for the duration of the semester.

#### Undergraduate TAs in lab

Undergraduate TAs in lab are the primary source of help for students working in the lab. They work regular weekly shifts covering the 60 hours/week that the lab is open, with two to four TAs available at all times. These TAs help students locate and use the materials and equipment needed for their projects, guide them through calculations and experimental protocols, provide troubleshooting assistance, and answer a wide range of questions. They also assist with lab maintenance activities. Undergraduate TAs meet weekly with the lab staff and course directors to discuss student projects, issues that arise in the lab, and for ongoing training.

#### Lab staff

Lab staff support the undergraduate TAs in the lab by helping to answer students’ questions when needed. The lab staff also maintain the lab space, manage the zebrafish facility, order supplies, and assist with other research-related activities.

#### Course directors

Course directors are responsible for the overall operation of the course, administratively and pedagogically. They design assignments and course activities, train TAs, manage the course Canvas sites and other electronic resources, provide advice, and grade some student work.

### Participants

Participants were undergraduate students enrolled in *Foundations of Biology for Biological Sciences Majors*, *Part II Laboratory* during the fall 2019 semester. Participants were recruited by email a few days prior to the start of the semester. Any interested students confirmed their interest in participating in the study and provided a phone number that could accept text messages through a web form. Using a random number generator [23], a maximum of six students per laboratory section from ten laboratory sections (two sections per research area) were randomly selected to participate (n = 57; see S1 Table for the number of participants by research area). In two laboratory sections, multiple attempts were made to recruit six participants, but fewer than six students were interested in participating in the study. The study participants (n = 57) represented 15.2% of the total course enrollment (N = 374). However, only 10 (out of 19) lab sections were randomly selected to participate in the study. Controlling for only participating lab sections, the study participants (n = 57) represented 30.2% of the total enrollment of the lab sections that were randomly selected to participate in the study (n = 189). The mean lab section enrollment was 20 (min = 12, max = 25). The University of Minnesota Institutional Review Board reviewed the research plans and granted an exempt status (Study 1405E50826). All students consented to participate and were free to opt out of the study. Participants were eligible to receive compensation at three time points during the semester (approximately once every five weeks). At each compensation time point, participants who completed at least 80% of the surveys during the previous five weeks received $25, either as an Amazon gift card or cash (participant’s choice).

### Data collection

Students’ perceptions of personnel resources were collected throughout the semester using two surveys—a text message survey and a web-based survey. Each survey was administered using the Qualtrics platform as described below.

#### Text message survey

Prior to receiving their first text message survey, students were texted an initial opt-in message to comply with legal and carrier requirements (S1 Appendix). This opt-in message stated who the following messages were coming from, what the messages were regarding, the phrase “standard messaging rates may apply”, and opt-out instructions. The opt-in message also included a link to a website for each laboratory section that included pictures of each personnel resource, their name, and their role (e.g., graduate TA). To ensure students knew who their resources were, the opt-in message also asked them to respond with the names of their research mentor and graduate TA. Nearly all respondents (89%) provided the correct names of both resources. Anyone who provided an incorrect response was sent an email with the correct response(s) and the link to their lab section’s personnel resources website. Some of the “incorrect” responses may have been due to the student changing lab sections during the add/drop window (prior to the second week of class).

The text message survey was designed to collect real-time data about students’ perceptions of personnel resources throughout their CRE (S2 Appendix). The survey was sent by text message 34 times throughout the semester, alternating between two or three times per week. The text message survey took less than five minutes to complete and collected data about which resource had been the most helpful since they last responded and how that resource was helpful. Students could respond to a text message survey until the next text message survey was sent out. The time between surveys ranged from two to four days (min 48 hours, max 96 hours).

#### Web-based survey

Since respondents could only provide information about a single personnel resource when responding to the text message survey, the web-based survey was designed to understand students’ perceptions of all personnel resources at a few time points throughout their CRE (S3 Appendix). The web-based survey was sent by email five times throughout the semester, approximately once every three weeks. The survey took five to ten minutes to complete and collected data about how frequently they used each resource and how helpful each resource was.

### Data inclusion and exclusion criteria

Of the 1432 text message survey responses, 55 (3.8%) were removed because either a resource was selected that was not available for their research area or the responses to who was the most helpful and how they were helpful did not agree (e.g., they selected graduate TA as the most helpful and when describing how they were helpful they used the name of the research mentor). The remaining text message responses (*N* = 1377) were included in the analysis. All of the web-based survey responses (*N* = 195) were included in the analysis.

### Quantitative data analysis

All data analysis was performed using R version 4.0.5 [24]. Response rates were calculated by dividing the number of respondents at each time point by the total number of study participants. Since response rates varied throughout the semester, all subsequent quantitative data (i.e., the most helpful resource, the frequency of resource use, and the level of resource helpfulness) are presented as the percentage of respondents that selected a particular response at each time point, not as the percentage of the total number of study participants. All reported percentages include responses from students across all research areas, some of which did not have all resources available (S2 Table). We focused on course-level data because the number of participants for each research area was small. Data by research area are provided as supporting information but should be interpreted with caution given the lower number of participants.

### Qualitative data analysis

Of the 1377 text message responses included in the analysis, 1284 responses included a response to the open-ended text message survey item asking how the resource they selected was helpful. These responses were categorized through multiple rounds of coding. A random selection of ten percent of the responses were used for the first rounds of coding, where initial coding was used to capture all possible reasons for how a resource was helpful [25]. Two coders (AO and SH) coded the responses independently, discussed codes, and grouped codes into categories with defined criteria (Table 1). The resulting categories were used to recode all previously coded responses and code the remaining responses. After coding all responses independently, the coders discussed any coding disagreements and came to a consensus. Initial coder percent agreement was 78.1% for complete agreement on all categories for a single response and 96.0% percent agreement across all categories for all responses. There were only two responses for which the coders could not come to a consensus about which categories the responses should be coded into; these two responses were not included in the analysis.

**Table 1:** Qualitative data analysis coding categories, criteria, and example responses.

Coding Category	Criteria	Example Responses
**Project-specific: feedback**	Includes responses that describe the resource providing feedback, answering questions, or helping in some way with the research project (e.g., help with choosing a research project topic; suggestions for or feedback on project-specific data visualizations or statistical analyses; feedback on papers, posters, or presentations)	*“My grad TA is a good resource for me to ask questions to regarding the research project topic I am in the process of choosing…”*
		*“She provided detailed feedback on our progress report and made sure we were on track for the project*.*”*
		*“We were able to ask questions*, *ask what was viable in our study design*, *ask which methods were better*, *and the research mentor knew what would be easiest for us to do in terms of our microbial study*.*”*
	Excludes responses that fit in the project-specific: troubleshooting or project-specific: resources categories.	
**Project-specific: troubleshooting**	Includes responses that describe the resource providing problem-solving help with technical aspects of projects (e.g., troubleshooting software or methods) or suggesting changes that would improve project quality or increase project efficiency	*“She helped me troubleshoot an error that kept coming up*.*”*
		*“*.* *.* *.* *.*in the lab our undergrad TA for my section really helped our group to figure out flukes we kept having with motion tracker/fish feeding*.*”*
		*“She helped us think of ways to improve our second experimental trial*.*”*
**Project-specific: resources**	Includes responses that describe the resource providing information about the location or availability of laboratory supplies (e.g., reagents, dishes, equipment, etc. for wet-lab projects; data sets, software, etc. for computational projects), how to perform procedures (e.g., laboratory techniques for wet-lab projects; coding for computational projects), how to use equipment or software, or multiple options for data visualization or statistical analysis	*“They got me the media I needed and supplied food*.*”*
		*“Directed us to people that might have data helpful to us”*
		*“He helped us with PCR and locating primers*.*”*
**General feedback**	Includes responses that describe the resource providing feedback, answering questions, or helping in some way that is not specific to the research project (e.g., responses that discuss work prior to the start of the research projects (lab skills training) or responses that are too general to know if it is specific to the research project)	*“She is able to answer my questions very thoroughly and knowledgeably*.*”*
		*“[Undergraduate TA in section] has been helping us out a ton with everything*.*”*
		*“She’s just good at keeping us on track*.*”*
**Positive attributes: general**	Includes responses that describe general positive personal qualities (e.g., nice)	*“He was very patient*.* *.* *.*”*
		*“My TA is so nice and generous*.*”*
**Positive attributes: approachable/ understanding**	Includes responses that describe the resource as being approachable or understanding	*“Very understanding and easy to talk to”*
		*“He is really open to questions and encourages us to come talk to him*.*”*
**Positive attributes: available/ responsive**	Includes responses that describe the resource as being available, responsive, or accessible	*“He gives prompt responses to emails*.*”*
		*“She has been accessible outside of class*.* *.* *.*”*
**Course logistics**	Includes responses that describe the resource providing information that would typically be found in a detailed course syllabus (e.g., setting assignment or behavioral expectations, timelines, due dates, poster session details)	*“Our grad TA sent out an email with things to complete for the week*.*”*
		*“My Grad TA was helpful because she described the requirements for the class and helped us understand the work required from us*.*”*
**Other instructional duties**	Includes responses that describe the resource performing other instructional duties (e.g., in-class instruction, grading, office hours or other meetings, attending the poster session)	*“She taught class*.*”*
		*“She graded our work*.*”*
		*“Is allowing me to meet with her for office hours”*

Qualitative data are reported as counts and percentages of each coding category within each resource. Coding category percentages were calculated as a percentage of the total number of responses for each resource that fell into that category, with each response having the potential to fall into more than one category (i.e., percentages can sum to greater than 100%).

## Results

### Survey response rates

**Fig 2:**
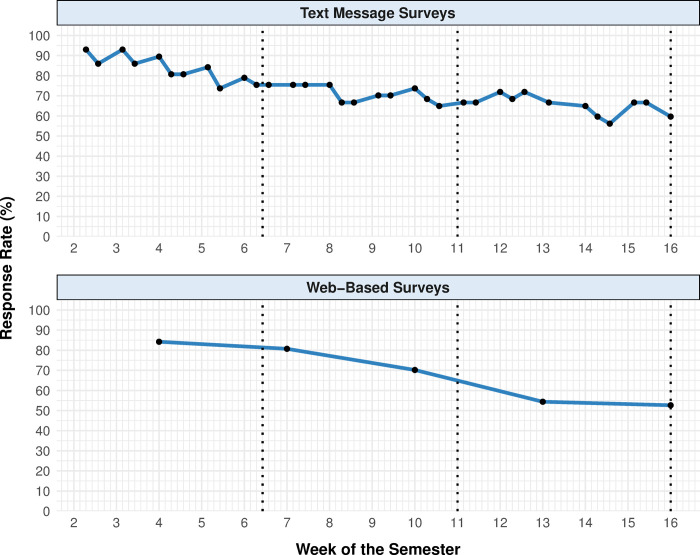
Survey response rates. Response rates are shown for text message surveys (top) and web-based surveys (bottom) throughout the semester. Vertical grid lines indicate days of the week beginning with Monday. Vertical dotted lines indicate the three dates when compensation was sent to respondents. Black points indicate the dates when the respective surveys were administered.

To understand which personnel resources students found valuable throughout a semester-long CRE, students (n = 57) were invited to respond to a text message survey 34 times (two or three times per week) and a web-based survey five times (once every three weeks) throughout the semester. The average response rate for the text message survey was 73.2% and ranged from 56.1% to 93.0% (Fig 2, top). The average response rate for the web-based survey was 68.4% and ranged from 52.6% to 84.2% (Fig 2, bottom). Project-specific text message survey and web-based survey response rates are provided in the supporting information (S1 Fig).

The majority of participants responded to most of the text message surveys; 40 participants (70.1%) responded to at least 24 (70.6%) text message surveys, and 36 participants (63.2%) responded to at least 27 (79.4%) of the text message surveys. The majority of participants also responded to most of the web-based surveys; 33 participants (57.9%) responded to at least four (80.0%) web-based surveys.

### Real-time student perceptions of personnel resources

By using a text message survey that was administered two or three times per week every week of the semester, we were able to collect real-time data about students’ perceptions of personnel resources which allowed us to investigate if students’ needs changed throughout their research projects. With each text message survey students were asked: 1) “Since your last response, which resource has been the most helpful?” and 2) “Please describe how the resource you chose was helpful.”

**Fig 3:**
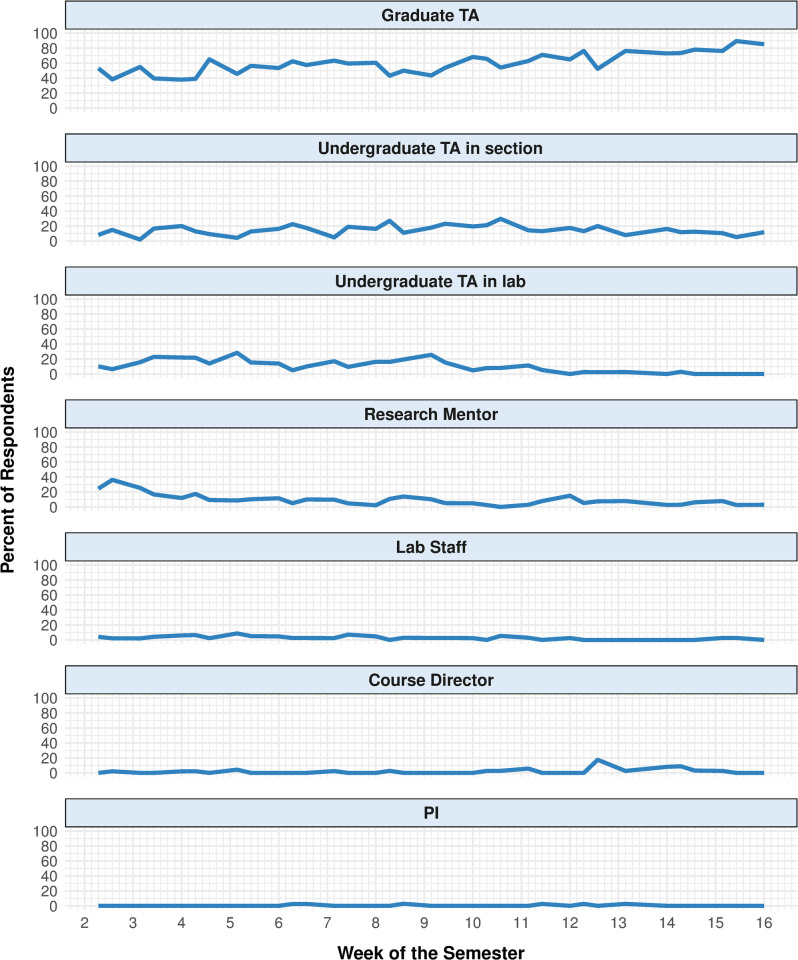
The most helpful resource. Percentage of respondents that selected each resource as “the most helpful resource” throughout the semester. Vertical grid lines indicate days of the week beginning with Monday.

Some resources were selected as the most helpful by a consistent percentage of the respondents throughout the semester, while others showed a time-dependent pattern (Fig 3). Throughout most of the semester, the majority of respondents selected graduate TAs as the most helpful resource (*M* = 60.2%). A lower, but consistent, percentage of respondents selected undergraduate TAs in section as the most helpful resources throughout the semester (*M* = 14.7%). Undergraduate TAs in lab were selected by a similar percentage of respondents as undergraduate TAs in section in the first half of the semester but were selected by very few respondents in the second half of the semester (*M* = 10.4%). Approximately 40% of the respondents selected research mentors as the most helpful resource in the second week of the semester, but that quickly trailed off to ~10% of the respondents for the remainder of the semester (*M* = 9.5%). A low, but consistent, percentage of the respondents selected lab staff throughout the semester (*M* = 2.7%). Course directors were selected by a low percentage of the respondents until the last few weeks of the semester when the percentage of respondents selecting them increased to ~20% (*M* = 2.0%). Throughout the semester, PIs were rarely selected as the most helpful resource (*M* = 0.5%). Project-specific response data for the most helpful resource are provided in the (S2 Fig).

Qualitative analysis of students’ descriptions for how a resource was helpful revealed a few themes, with most responses describing resources helping by providing project-specific feedback, general feedback, or assistance obtaining project-specific resources (e.g., lab supplies). The full list of coding categories, a description of the criteria, and example responses are shown in Table 1. Counts and percentages for the coding of responses associated with each resource are shown in Table 2. Overall semester trends are reported for the qualitative analysis instead of a weekly breakdown because the types of support students described for each resource were consistent throughout the semester.

**Table 2:** Qualitative coding counts and percentages for how each resource was helpful.

Coding Category	Graduate	Undergraduate	Undergraduate	Research	Lab	Course	PI
	TA	TA in section	TA in lab	Mentor	Staff	Director	
	*N* = 754	*N* = 185	*N* = 144	*N* = 137	*N* = 35	*N* = 24	*N* = 5
**Project-specific: feedback**	460 (61%)	53 (29%)	14 (10%)	87 (64%)	3 (9%)	13 (54%)	1 (20%)
**Project-specific: troubleshooting**	44 (6%)	31 (17%)	19 (13%)	10 (7%)	5 (14%)	1 (4%)	0 (0%)
**Project-specific: resources**	72 (10%)	46 (25%)	62 (43%)	37 (27%)	19 (54%)	3 (12%)	3 (60%)
**General feedback**	134 (18%)	62 (34%)	57 (40%)	19 (14%)	9 (26%)	1 (4%)	2 (40%)
**Positive attributes: general**	31 (4%)	9 (5%)	5 (3%)	2 (1%)	1 (3%)	1 (4%)	0 (0%)
**Positive attributes: approachable/ understanding**	5 (1%)	0 (0%)	1 (1%)	1 (1%)	0 (0%)	0 (0%)	0 (0%)
**Positive attributes: available/responsive**	31 (4%)	13 (7%)	7 (5%)	4 (3%)	0 (0%)	1 (4%)	0 (0%)
**Course logistics**	67 (9%)	10 (5%)	1 (1%)	2 (1%)	1 (3%)	4 (17%)	0 (0%)
**Other instructional duties**	45 (6%)	0 (0%)	2 (1%)	15 (11%)	0 (0%)	7 (29%)	0 (0%)

Values are n (%). The blue shading corresponds to the percentage of responses within each coding category where lighter blue indicates a lower percentage of responses and a darker blue indicates a higher percentage of responses. The shading darkens in 20% increments.

#### Graduate TAs

The majority of responses describing how graduate TAs were helpful said they provided research project-specific feedback, which included things like helping with choosing a research project topic or providing feedback on their paper, poster, or presentation. For example:

- *‘He is a good resource for getting help regarding the research topic that has been narrowed down to be more accurate*.*’*
- *‘Gave us feedback on our poster as well as described how to write the abstract and discussion portions of our paper’*
- *‘Provided feedback on our poster that prepared me for our presentation’*

#### Undergraduate TAs

Undergraduate TAs were described as helping in a variety of ways and did not have a majority of their responses fall into one coding category. Approximately one third of responses for undergraduate TAs in section described them providing project-specific feedback (e.g., *‘She gave our group constructive feedback on our poster’*). One quarter of responses described undergraduate TAs in section providing project-specific resources, which included providing information about the location of laboratory supplies, how to perform procedures, or how to use equipment or software. For example:

- *‘They gave us plates*.*’*
- *‘Told us how to go about creating stock culture tubes with parafilm’*
- *‘She helped us run our code*.*’*

In addition, one third of responses for undergraduate TAs in section said they provided general feedback, which included any feedback that was not related to the research project or responses that were too general to know if they pertained to the research project. For example:

- *‘She really helped us when we were struggling in lab*!*’*
- *‘He stayed after class to help us with a problem*.*’*
- *‘Answered all questions asked’*

Similarly, responses for undergraduate TAs in lab were fairly evenly divided between providing project-specific resources (e.g., *‘She helped me find reagents and determine what I should do*.*’*) and general feedback (e.g., *‘He helped me in lab and answered any questions we had*.*’*), with approximately 40% of responses falling into each category.

#### Research mentors

The majority of responses for research mentors described them providing research project-specific feedback (e.g., *‘The research mentor helped us narrow down our experiment and its hypothesis into something that can easily be supported or not*. *Prior to this*, *our hypothesis was broad and difficult to analyze*.*’*). Approximately one quarter of responses for research mentors described them as providing project-specific resources (e.g., *‘When we explained our project to him*, *he gave us extremely helpful tips on the best way to do our procedure in the amount of time we had*. *He really was able to fill in the blanks in our project*. *We weren’t sure on some aspects of how to choose different concentrations of chemicals and he gave us procedures in a detailed yet easily understandable way*.*’)*

#### Lab staff

The majority of responses for lab staff described them providing project-specific resources (e.g., *‘Helped us get the chemicals we need to run our experiment’*). In addition, 26% of responses for lab staff described them providing help through general feedback (e.g., *‘Offer a lot of help’*).

#### Course directors

The majority of responses describing how course directors were helpful said they provided research project-specific feedback (e.g., *‘She gave feedback on the poster*.’). Approximately one third of responses for course directors described them as providing help through other instructional duties (e.g., in-class instruction, grading, office hours, attending poster session). For example:

- *‘She graded our work*.*’*
- *‘They were at the poster session*.*’*

#### PIs

The majority of responses for PIs described them providing project-specific resources (e.g., *‘Helped us find our materials’*). In addition, 40% of responses described PIs providing help through general feedback (e.g., *‘He said what we were doing good and how to add better things on*.*’*).

### Retrospective student perceptions of personnel resources

While the text message surveys provided real-time data of students’ perceptions of personnel resources, respondents were only able to provide information about one resource at each time point. We used a web-based survey to understand students’ perceptions of all resources at a few time points throughout the semester. The web-based survey asked students to indicate: 1) how often they used each of the resources using a five-point scale ranging from “Never” to “Always” and 2) how helpful each of the resources had been using a five-point scale ranging from “Not at all” to “Extremely”.

**Fig 4:**
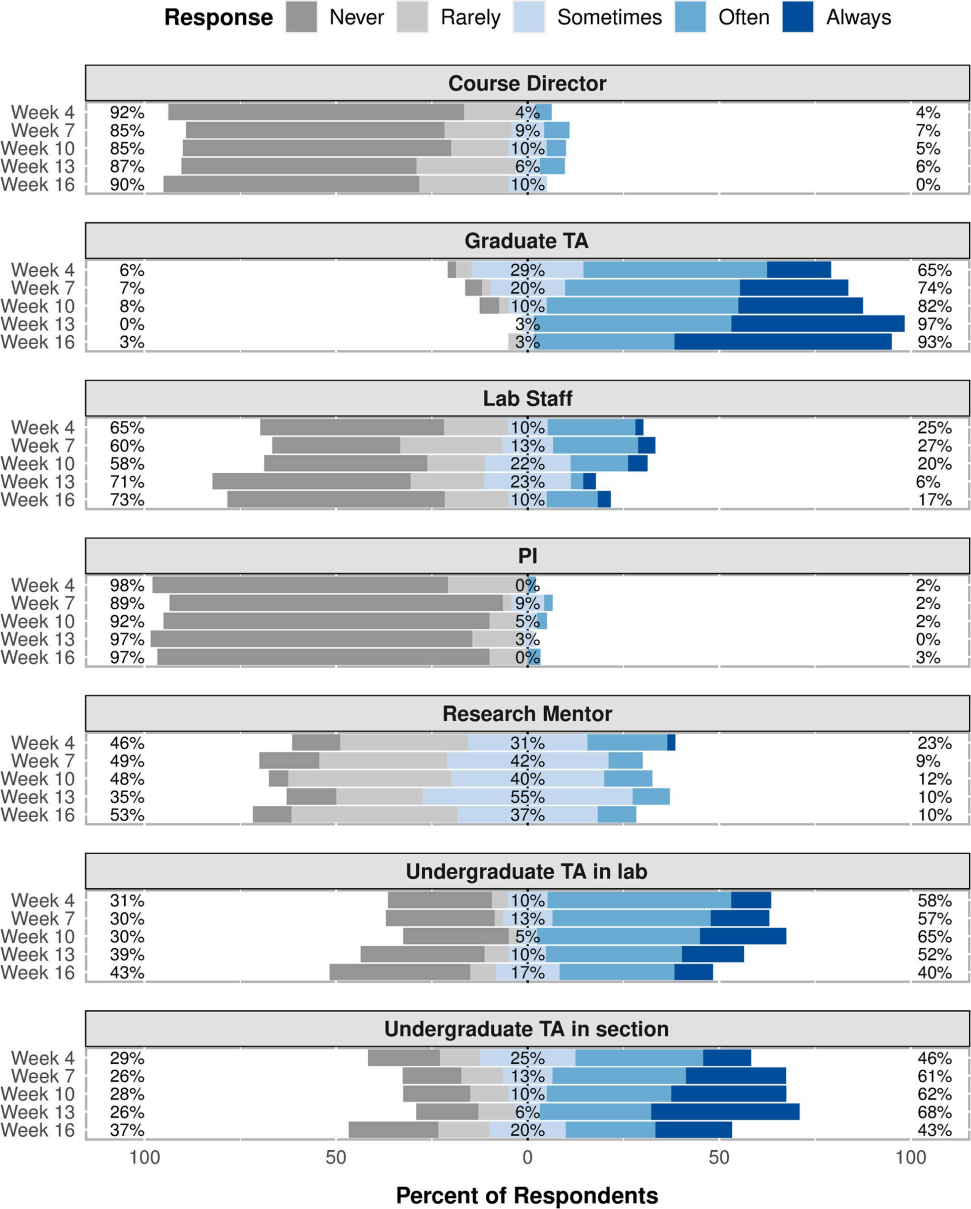
Resource use frequency. Frequency that respondents reported using each resource at five time points throughout the semester. The percentages on the left side of the chart are the sum of the “Never” and “Rarely” response percentages. The “Sometimes” response percentages are shown in the middle of the chart. The percentages on the right side of the chart are the sum of the “Often” and “Always” response percentages.

The frequency that respondents reported using each resource largely mirrors the results of the text message survey, though some time-dependent trends seen in the text message survey results were not seen in the web-based survey results (Fig 4). Throughout the semester, the majority of respondents reported using graduate TAs “Often” or “Always”, with the percentage of respondents increasing from 65% at the beginning of the semester to 93% at the end of the semester. The percentage of respondents who reported using undergraduate TAs in section “Often” or “Always” varied throughout the semester from 43% to 68%. A larger percentage of respondents (~60%) reported using undergraduate TAs in lab “Often” or “Always” in the first half of the semester compared to the end of the semester (40%). Research mentors were used “Often” or “Always” by ~20% of the respondents in the beginning of the semester, but only ~10% of respondents used them at that frequency for the remainder of the semester. The percentage of respondents who reported using lab staff “Often” or “Always” varied throughout the semester from 6% to 27%. Throughout the semester, only a small percentage of respondents reported using course directors (~5%) or PIs (~2%) “Often” or “Always”.

**Fig 5:**
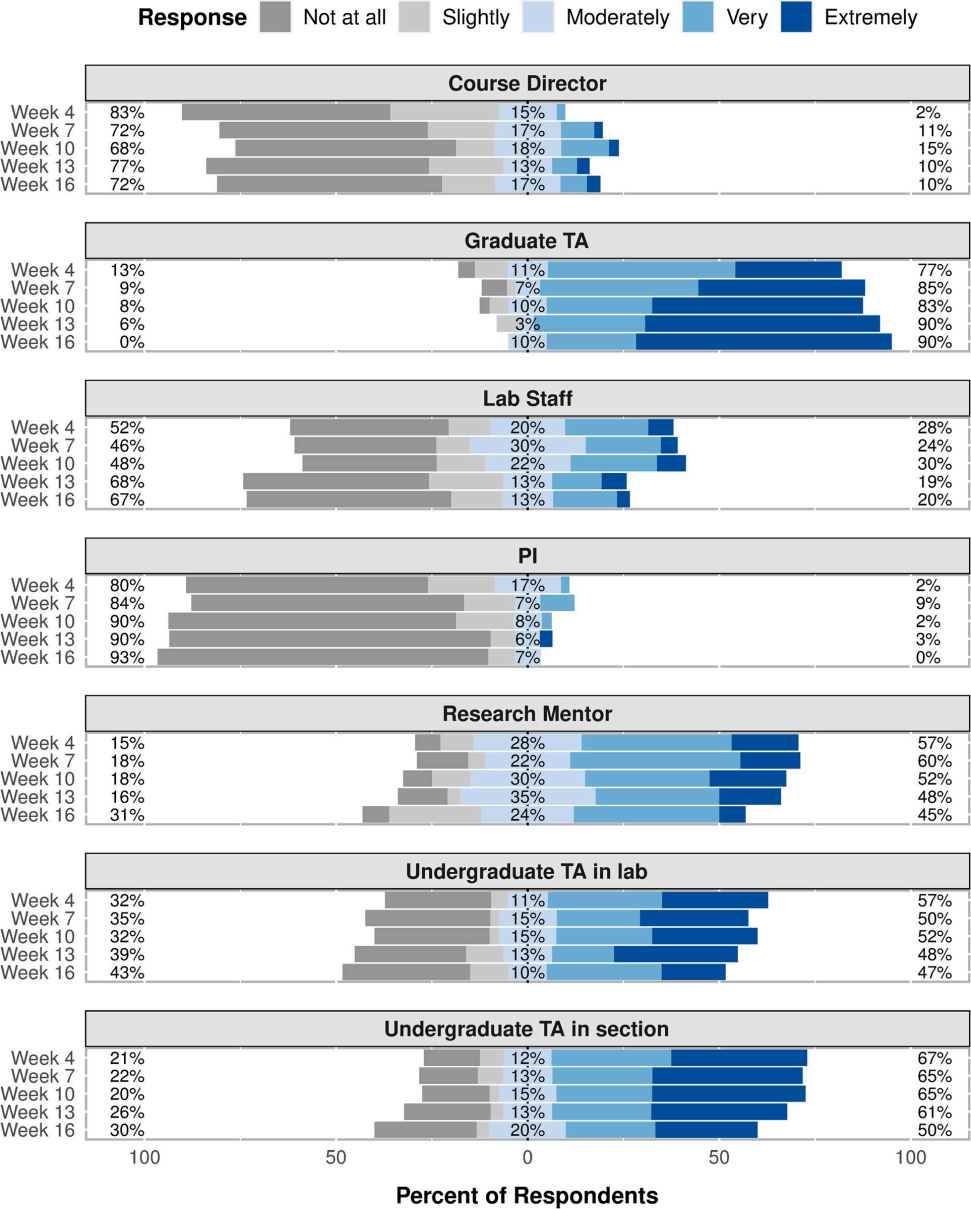
Resource level of helpfulness. Level of helpfulness that respondents reported for each resource at five time points throughout the semester. The percentages on the left side of the chart are the sum of the “Not at all” and “Slightly” response percentages. The “Moderately” response percentages are shown in the middle of the chart. The percentages on the right side of the chart are the sum of the “Very” and “Extremely” response percentages.

The level of helpfulness that respondents reported for each resource also largely mirrors the results of the text message survey, though, again, some time-dependent trends seen in the text message survey results were not seen in the web-based survey results (Fig 5). Throughout the semester, the majority of respondents (77–90%) reported that graduate TAs were “Very” or “Extremely” helpful. A smaller majority of respondents (~65%) also reported that undergraduate TAs in section were “Very” or “Extremely” helpful throughout most of the semester. Approximately 50% of respondents reported that undergraduate TAs in lab were “Very” or “Extremely” helpful throughout the semester. The percentage of respondents who reported that research mentors were “Very” or “Extremely” helpful varied throughout the semester from 45% to 60%. The percentage of respondents who reported that lab staff were “Very” or “Extremely” helpful was slightly larger in the first half of the semester (~30%) compared to later in the semester (~20%). Throughout the semester, only a small percentage of respondents reported that course directors (~10%) or PIs (~3%) were “Very” or “Extremely” helpful.

## Discussion

In this study, we used two survey methods to collect data about students’ perceptions of personnel resources during a CRE—a frequent, brief text message survey and a less frequent, more comprehensive web-based survey. Our use of the text message survey was a novel approach for understanding the students’ experience in a CRE setting and had many benefits over a web-based survey approach. First, we were able to collect real-time data about students’ perceptions at multiple time points throughout their research experience, rather than relying on students to accurately recall their experiences later. Without the frequent text message surveys, we may have missed the temporal variability of students’ perceptions of some personnel resources (e.g., research mentors were most helpful at the beginning of the semester, course directors were most helpful towards the end of the semester). Second, the response rates were higher for the text message surveys than the web-based surveys even though students were asked to complete the text message surveys multiple times per week vs. a few times a semester for the web-based surveys. Taken together, not only did the text message survey method provide more accurate data than the web-based survey, it also provided a more complete data set. The disadvantage to the text message survey was in its brevity. By limiting the text message survey to a few questions, we were only able to collect students’ perceptions about a single personnel resource each time the survey was administered. In contrast, the web-based survey contained matrixed, Likert-type questions which allowed us to collect students’ perceptions about all personnel resources each time the survey was administered. Combining both methods allowed for a more complete understanding of students’ perceptions of personnel resources.

Our findings from these surveys address three key questions:

### 1. Which personnel resources were the most valuable to students during the CRE?

Most salient from student reports—both text message and web-based surveys—was the overwhelming importance of the graduate TAs, and, to a lesser extent, undergraduate TAs for the CRE. Graduate TAs are most often cited as the most helpful resource from the days preceding each text message request, and they are ranked as “very helpful” or “extremely helpful” by the majority of students at each web-based survey timepoint. This finding aligns well with prior reports, which attest to the positive impact of graduate TAs on, for example, student performance and persistence in a discipline [26–28]. In fact, only the undergraduate TAs—those staffing the lab and those assigned to the lab section—approached the perceived helpfulness of graduate TAs. These three categories are visible, consistent sources of help throughout the CRE, and student comments about their helpfulness affirm this. TAs give project-specific and general feedback, and identify project-specific resources that help students complete their CRE work (Table 2).

The course directors and project PIs were the least cited as the most helpful in the preceding days. We hesitate to draw firm conclusions about their actual utility from these data, however, in that their work may constitute much of the “shadow” effort associated with the implementation of CREs. For example, the course directors help graduate TAs prepare for teaching each week, manage peer reviews during the writing process, and organize the poster sessions where students present their projects, but students are often unaware of this work. Similarly, the project PI may have conceived of the original project for a CRE, gotten the funding to obtain the data (e.g., especially in the case of computational CREs), or trained the research mentor. These are important functions that support the development of a CRE, but may not be appreciated by the students. However, these findings do cast doubt on how critical PI presence, and the need for PI presence to establish the work as broadly relevant, actually is for CRE implementation; this is an issue that has been debated in other studies, including some from our team [8, 29, 30]. Perhaps the research mentor could be counted on to assume this role in the CRE, giving students access to a legitimate member of the research team, without imposing the burden—and possibly the barrier—of needing the involvement of a committed PI.

Student impressions of the lab staff and research mentors occupy a middle ground in our ranking of the helpfulness of CRE personnel. Research mentors were more often cited as the most helpful personnel early in the semester, when students were planning their projects, and cited at lower, more variable frequencies later in the semester. Lab staff (for the lab-based research areas) had more consistent, but fewer, citations from students. As with course directors and PIs, some of the effort by these individuals, such as research mentors answering questions from graduate TAs and lab staff supporting undergraduate TAs, may not be obvious to students.

### 2. In what ways were the personnel resources valuable to students?

Qualitative analysis of students’ open-ended responses generally fell into two large themes—project-specific help or feedback and general help or feedback (Table 2). Student comments largely affirmed that personnel were doing the jobs they were assigned to do for the CRE. Course directors graded work, lab staff helped acquire chemicals, and undergraduate TAs gave constructive feedback during poster development. Students had the most to say about the many ways in which the graduate TAs were helpful; these included helping the students narrow their research questions, write their final papers, and prepare their final presentations. It is worth noting that undergraduate TAs provided somewhat different types of help than graduate TAs: often they gave general feedback, or more practical help with resources or troubleshooting, rather than the project-specific feedback provided by graduate TAs or research mentors. In particular, undergraduate TAs in section handle questions that fit with their expertise, freeing the graduate TAs to focus on broader scientific issues and project-specific feedback. The themes associated with each resource did not vary throughout the semester.

### 3. Were different personnel resources more valuable to students at different times during the CRE (e.g., during the project planning at the beginning of the semester vs. during the presentation of results at the end of the semester)?

There was some temporal variability in student perceptions of human resources (Fig 3). For example, students were more likely to cite graduate TA help with posters during the end of the term, when they were actually making their posters for a final poster session. Furthermore, the overall helpfulness of graduate TAs seemed to only increase over the semester. That of the research mentors may have decreased a bit, once the student research was underway. For other personnel, student impressions seemed fairly consistent (and unremarkable). In sum, students may rely on different resources at different times in the research project, but the type of support a particular resource provides does not change much throughout the research project. Table 3 summarizes student activities during a CRE and the type of support students cited as helpful during those activities.

**Table 3:** Summary of research project activities and student-cited support needed during each activity.

Activity	Support
Research project planning	Feedback on project topic and proposed methodology,
	Assistance finding/ordering supplies
Research project work	Assistance finding/ordering supplies,
	Assistance operating/troubleshooting equipment,
	Assistance performing/troubleshooting procedures,
	Assistance revising plans (if needed),
	Assistance writing/troubleshooting code
Data analysis, making figures	Assistance choosing/performing statistical methods,
	Assistance using statistical software,
	Assistance creating or choosing appropriate figures,
	Assistance writing/troubleshooting code
Designing and presenting posters	Feedback on what to include in a poster, how to format a poster,
	and how to present a poster
Writing research paper	Guidance on how to write different sections of a research paper,
	Feedback on drafts

Although not part of our original research aims, we do note that students’ perceptions and use of personnel resources is similar across most research areas we investigated, especially with respect to the consistently high percentage of respondents that selected graduate TAs as the most helpful resource throughout the semester (S2 Fig). The exception to this trend was seen in only one research area—microbial evolution—where an approximately equal percentage of respondents selected graduate TAs and undergraduate TAs as the most helpful resource throughout the semester. This difference might be related to the personalities of the TAs and/or students in these two sections.

There are some limitations to our study. First, this study examines students’ perceptions of how personnel are valuable to them, but students are often not aware of the roles of personnel outside of the classroom and lab, as noted above. Second, all personnel resources were not available in all research areas, however, all research areas had graduate TAs, undergraduate TAs in section, research mentors, and course directors. Therefore, the main conclusions we draw from this study—especially the critical role of graduate TAs in these CREs—is not affected by this limitation. Third, the familiarity of the personnel resource (i.e., a resource students interact with on a weekly basis vs. a resource students interact with less frequently) was not accounted for in our analysis. Finally, while this was designed to be a descriptive study, our sample size and context (single course, single semester) limit the broad generalizability of the results.

Despite these limitations, this work represents important foundational information regarding the experiences of students within CREs. As the prevalence of CREs continues to grow, it is imperative that instructors have an understanding how students may use different types of human and material resources such that they can design their course to best support students within the operational constraints they may have. In this manner, our work attempts to shed light on a previously understudied facet of CRE design rather than produce a highly generalizable description of student resource use across the highly diverse landscape of CRE designs and implementations. As in many disciplines across the biological sciences, descriptive studies have a key place within biology education research [31] particularly in establishing a foundation from which more expansive quantitative and comparative studies can be conducted. The study presented here clearly identifies some key areas for future work that would benefit from a comparative approach. For example, how do instructor or TA characteristics (such as gender identity, international status, primary language spoken, race/ethnicity etc.) impact students’ perception of their value? Similarly, one could ask questions about how the level of teaching support, experience, or professional development of an instructor impacts students’ perception of value. While these are certainly critical additional questions to answer, the inability of this study to answer them in a generalizable way does not inherently limit the value of this work. Science is built upon the incremental increase in understanding and here we provide a descriptive analysis of the student experience in one institution as a building block for better understanding how to effective designs CREs for undergraduate students.

## Conclusions

In sum, graduate and undergraduate TAs serve a critical function when it comes to these research experiences. This finding is not necessarily surprising, but it raises the question of whether graduate and undergraduate TAs are actually prepared to facilitate inquiry effectively. The training of TAs is an area in need of scrutiny, especially as so many of the current exhortations for reform (e.g., inquiry-based instruction) are focused on the environment of the teaching laboratory and thus place an added burden on TAs. However, little work has focused on training TAs to facilitate inquiry [32–34], and TA training programs appear to vary a great deal [34–38]. Our findings simply highlight the need for departments and course coordinators to be deliberate and thoughtful in planning TA training that prepares TAs to assume the critical positions they occupy in CRE implementation. For example, some of our earlier work has highlighted the importance of having TAs *model* facilitation strategies as part of their training—as opposed to just *telling* them what to do [33]. Of course, before we—or anybody—can prescribe specific training methods, further research is needed into how best to train TAs to facilitate inquiry.

## Supporting information

S1 Fig Project-specific survey response rates. Project-specific response rates are shown for text message surveys (top) and web-based surveys (bottom) throughout the semester. Vertical grid lines indicate days of the week beginning with Monday. Vertical dotted lines indicate the three dates when compensation was sent to respondents.
(TIF)

S2 Fig The most helpful resource by project. Percentage of respondents that selected each resource as “the most helpful resource” throughout the semester by project. Vertical grid lines indicate days of the week beginning with Monday.
(TIF)

S1 Table Number of participants by research area. (PDF)

S2 Table Resources available for each research area. (PDF)

S1 Appendix Initial text message survey. (PDF)

S2 Appendix Text message survey. (PDF)

S3 Appendix Web-based survey. (PDF)
