# Supplementary figures and images for: Real-time text message surveys reveal student perceptions of personnel resources throughout a course-based research experience
Source: PLoS One. 2022 Feb 18;17(2):e0264188. doi: 10.1371/journal.pone.0264188 (PMC8856569; doi:10.1371/journal.pone.0264188)

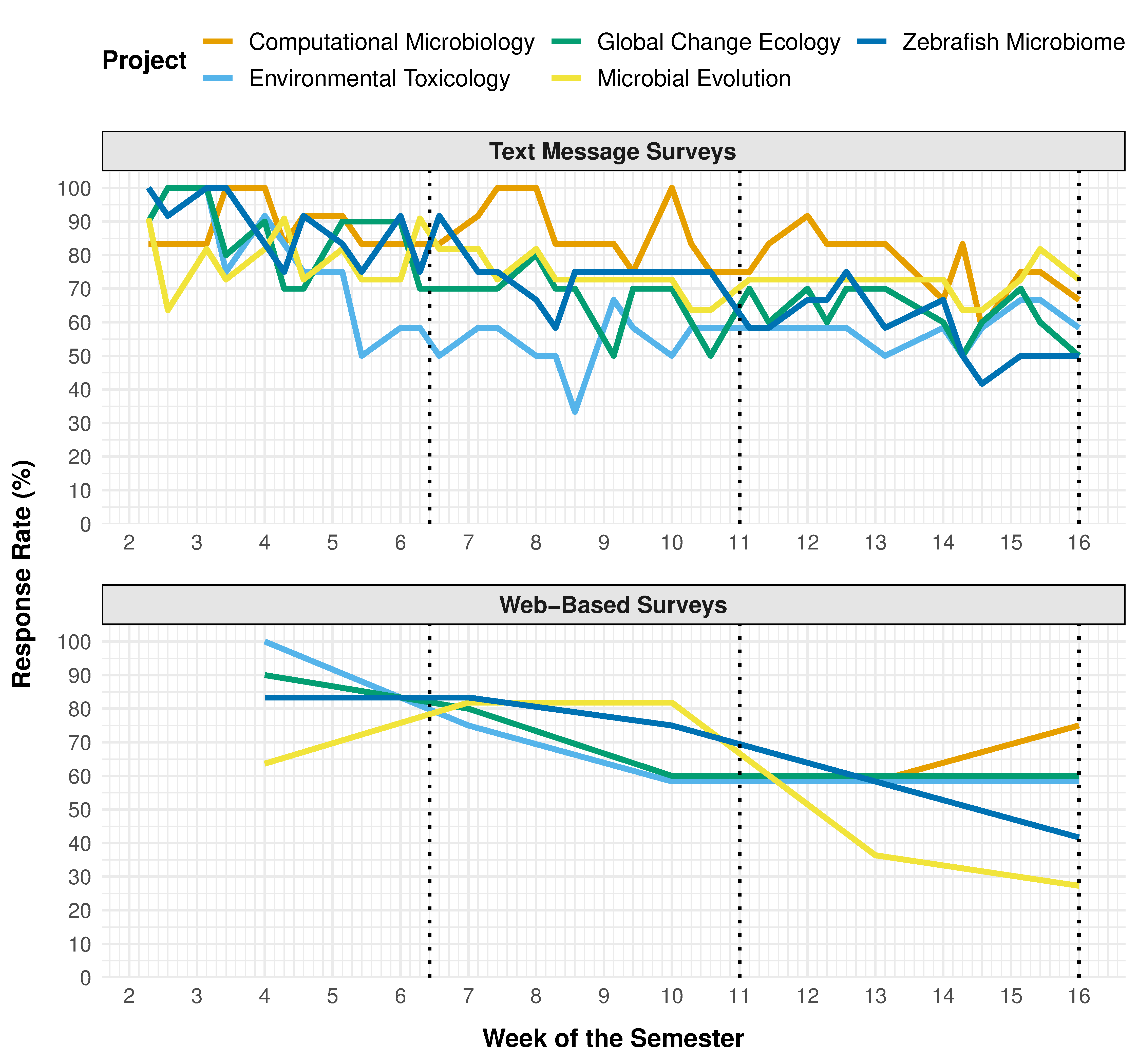

Supplement: S1 Fig — Project-specific response rates are shown for text message surveys (top) and web-based surveys (bottom) throughout the semester. Vertical grid lines indicate days of the week beginning with Monday. Vertical dotted lines indicate the three dates when compensation was sent to respondents. (TIF) [file pone.0264188.s001.tif]

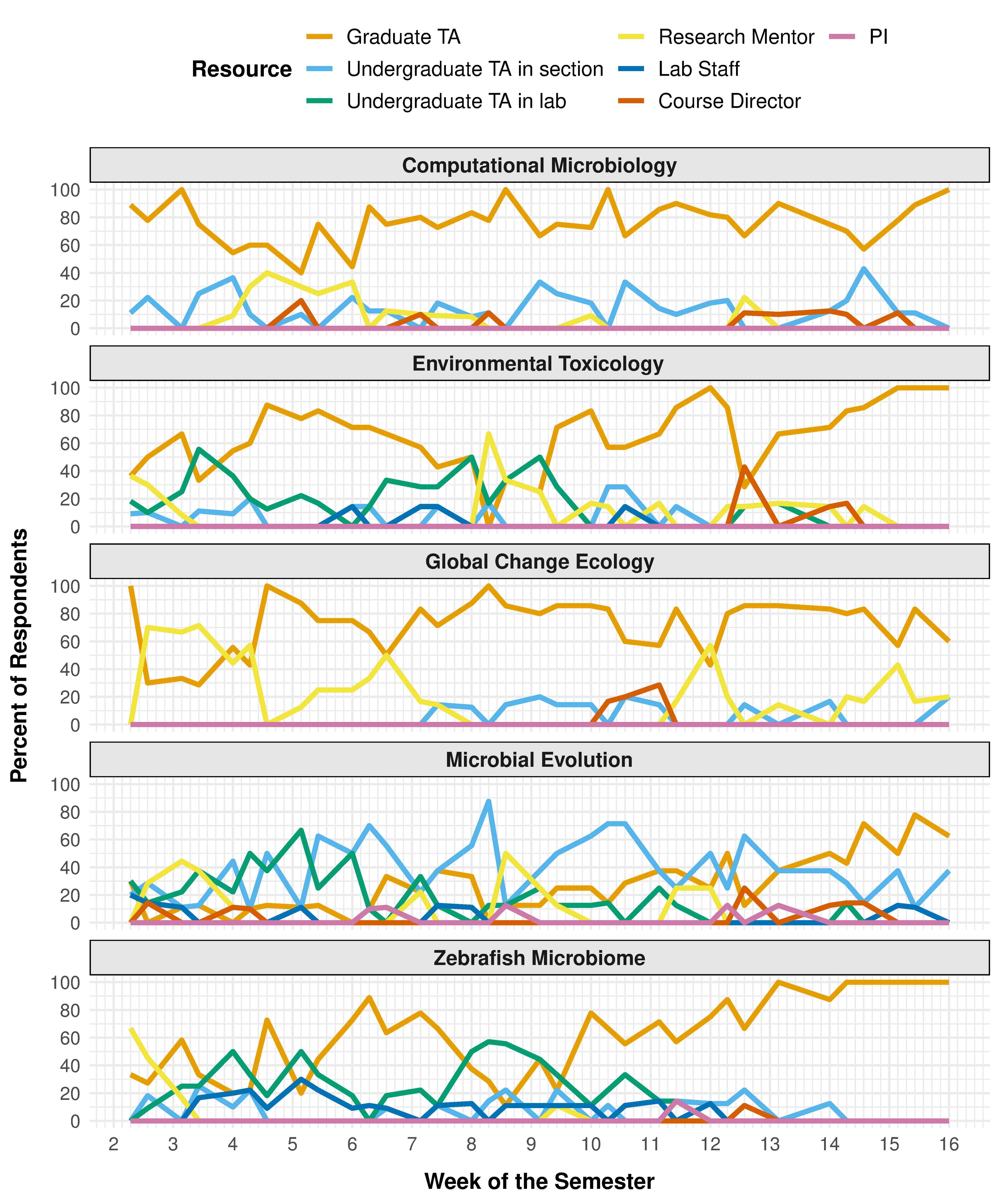

Supplement: S2 Fig — Percentage of respondents that selected each resource as “the most helpful resource” throughout the semester by project. Vertical grid lines indicate days of the week beginning with Monday. (TIF) [file pone.0264188.s002.tif]
