# Supplementary material for: Real-time text message surveys reveal student perceptions of personnel resources throughout a course-based research experience
Source: PLoS One. 2022 Feb 18;17(2):e0264188. doi: 10.1371/journal.pone.0264188 (PMC8856569; doi:10.1371/journal.pone.0264188)
Supplement: S1 Table — (PDF) [file pone.0264188.s003.pdf]

S1 Table. Number of participants by research area.

Research Area	*N*
Computational microbiology	12
Environmental toxicology	12
Global change ecology	10
Microbial evolution	11
Zebrafish microbiome	12
