# Supplementary material for: Real-time text message surveys reveal student perceptions of personnel resources throughout a course-based research experience
Source: PLoS One. 2022 Feb 18;17(2):e0264188. doi: 10.1371/journal.pone.0264188 (PMC8856569; doi:10.1371/journal.pone.0264188)
Supplement: S2 Table — (PDF) [file pone.0264188.s004.pdf]

S2 Table. Resources available for each research area.

	Graduate TA	Undergraduate TA in section	Undergraduate TA in lab	Research Mentor	Lab Staff	Course Director	PI
Computational microbiology	X	X	NA	X	NA	X	NA
Environmental toxicology	X	X	X	X	X	X	NA
Global change ecology	X	X	NA	X	NA	X	X
Microbial evolution	X	X	X	X	X	X	X
Zebrafish microbiome	X	X	X	X	X	X	X
