# Supplementary material for: Real-time text message surveys reveal student perceptions of personnel resources throughout a course-based research experience
Source: PLoS One. 2022 Feb 18;17(2):e0264188. doi: 10.1371/journal.pone.0264188 (PMC8856569; doi:10.1371/journal.pone.0264188)
Supplement: S1 Appendix — (PDF) [file pone.0264188.s005.pdf]

S1 Appendix. Initial text message survey.

The following messages are from the Dept of Biology Teaching and Learning regarding your BIOL 3004 research project. Standard messaging rates may apply. Text STOP to opt out of this survey.

Review your BIOL 3004 resources:
[link to webpage (not public)]

Question 1
Who is your Research Mentor?

Question 2
Who is your Grad TA?
