# Supplementary material for: Real-time text message surveys reveal student perceptions of personnel resources throughout a course-based research experience
Source: PLoS One. 2022 Feb 18;17(2):e0264188. doi: 10.1371/journal.pone.0264188 (PMC8856569; doi:10.1371/journal.pone.0264188)
Supplement: S2 Appendix — (PDF) [file pone.0264188.s006.pdf]

S2 Appendix. Text message survey.

Question 1
Since your last response, which resource has been the most helpful?
1-PI
2-Research Mentor
3-Grad TA
4-Undergrad TA section
5-Undergrad TA lab
6-Lab staff
7-Course Dir.

Question 2
Please describe how the resource you chose was helpful.
