# Supplementary material for: Real-time text message surveys reveal student perceptions of personnel resources throughout a course-based research experience
Source: PLoS One. 2022 Feb 18;17(2):e0264188. doi: 10.1371/journal.pone.0264188 (PMC8856569; doi:10.1371/journal.pone.0264188)
Supplement: S3 Appendix — (PDF) [file pone.0264188.s007.pdf]

S3 Appendix. Web-based survey.

Question 1

How often have you used the following resources?

	**Never**	**Rarely**	**Sometimes**	**Often**	**Always**
PI	○	○	○	○	○
Research mentor	○	○	○	○	○
Graduate TA	○	○	○	○	○
Undergraduate TA in section	○	○	○	○	○
Undergraduate TA in lab	○	○	○	○	○
Laboratory staff	○	○	○	○	○
Course director	○	○	○	○	○

Question 2

How helpful have each of the following resources been?

	**Not at all**	**Slightly**	**Moderately**	**Very**	**Extremely**
PI	○	○	○	○	○
Research mentor	○	○	○	○	○
Graduate TA	○	○	○	○	○
Undergraduate TA in section	○	○	○	○	○
Undergraduate TA in lab	○	○	○	○	○
Laboratory staff	○	○	○	○	○
Course director	○	○	○	○	○
